# A CO_2_-switchable amidine monomer: synthesis and characterization

**DOI:** 10.1080/15685551.2016.1270027

**Published:** 2016-12-26

**Authors:** Hanbin Liu, Hongyao Yin, Yujun Feng

**Affiliations:** ^a^ Chengdu Institute of Organic Chemistry, Chinese Academy of Sciences, Chengdu, P.R. China; ^b^ State Key Laboratory of Polymer Materials Engineering, Polymer Research Institute, Sichuan University, Chengdu, P.R. China

**Keywords:** CO_2_-switchable, smart polymer, monomer, synthesis, stimuli-responsive

## Abstract

Smart system employed CO_2_ gas as new trigger has been attracting enormous attention in recent years, but few monomers that are capable of switching their hydrophobicity/hydrophility upon CO_2_ stimulation have been reported. A novel CO_2_ responsive monomer, 4-vinylbenzyl amidine, is designed and synthesized in this work with *N,N*-dimethylacetamide dimethyl acetal and 4-vinylbenzyl amine that is prepared through the Gabriel reaction. In bi-phase solvent of *n*-hexane and water, the monomer dissolves in *n*-hexane first and then transforms into water upon the CO_2_ treatment, indicating a hydrophobic to hydrophilic transition. This transformation is demonstrated as reversible by monitoring the conductivity variation of its wet dimethyl formamide solution during alternate bubbling/removing CO_2_. The protonation of 4-vinylbenzyl amidine upon CO_2_ treatment is demonstrated by ^1^H NMR which also accounts for the dissolubility change. The reversible addition-fragmentation chain-transfer polymerization of this monomer is also performed, finding the reaction only occurs in glacial acetic acid. The reason can be ascribed to the different radical structure produced in different solvent.

## Introduction

CO_2_ as a new trigger for smart systems has various advantages over its traditional counterparts such as pH, temperature, redox, light and voltage [1–4]. The first virtue is the good switchabilility, which means the responsive process can be repeated for much more times without significant attenuation or any contamination into the system [5]. The easy-removing feature of the gas state of CO_2_ may account for this characteristic. Furthermore, the CO_2_ is a metabolite of living cells rendering it good biocompatibility [6,7]. Last but not the least, CO_2_ is a waste gas with wide availability and low price. All these advantages push CO_2_-responsive compounds and polymers into the spotlight [1].

Jessop team [5] first reported a long-chain alkyl amidine compound which worked as a CO_2_-switchable surfactant that can stabilize emulsion under exposing of CO_2_ and break the emulsion by bubbling inert gas in higher temperature. Naturally, researchers want to figure out what would happen if the amidines groups are introduced into polymers. Some efforts have been made to achieve CO_2_-responsive polymers by post modification. Take our previous work as an example, we prepared poly(4-chloromethylstyrene) (PCMS) first by reversible addition-fragmentation chain transfer (RAFT) polymerization. Then the chloro groups were transferred into azido units in the second step. Finally, a homemade N′-propargyl-N,N-dimethylacetamidines (PDAA) were coupled with the azido units through a ‘click’ chemical process, yielding amidine-containing polymers [8,9]. Lowe and coworkers [10] synthesized polymers containing pentafluorophenyl acrylate (PFPA) and then substituted the pentafluorophenyl groups with amidine species histamine (HIS), resulting in amidine side chains. Both of these methods can target CO_2_-responsive polymers efficiently, but they seem tedious involving with functionalization after polymerization. Yuan and his coworkers [11,12] first synthesized monomer (N-amidino)dodecyl acrylamide (AD) that can be switched by CO_2_ and developed ‘breathable’ vesicles with its block copolymer. Subsequently, similar acrylamide monomers containing amidine groups were synthesized with same route as well [13,14]. Meanwhile, the CO_2_-sensitivity of commercial available monomers containing tertiary amine and acid groups were discovered by Zhao’s team [15–18]. However, the CO_2_-responsive monomer is rarely reported to date, resulting in limited choice can be made to fabricate CO_2_-sensitive polymers.

**Scheme 1.**
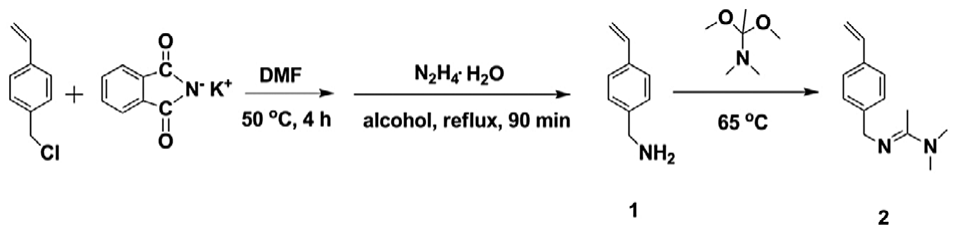
Synthesis route of 4-vinylbenzyl amidine.

Here in this work, an amidine monomer that is responsive to CO_2_ is developed. 4-Vinylbenzyl chloride was first transfer into 4-Vinylbenzylamine through the Gabriel reaction. Then a CO_2_-responsive monomer, 4-vinylbenzyl amidine, is produced after a followed reaction of 4-Vinylbenzylamine with *N,N*-Dimethylacetamide dimethyl acetal (Scheme 1). The CO_2_-responsive feature of this monomer is demonstrated by visualizing the hydrophobility to hydrophility transition and monitoring the conductivity variation as well as NMR shifts of its solution under CO_2_-stimulation. The discovery of this work paves the way to development of new CO_2_-sensitive polymer materials.

## Experimental part

### Materials and characterization

4-Vinylbenzyl chloride (90%), phthalimide potassium salt (99%), hydrazine monohydrate (N_2_H_4_·H_2_O, 50%), KOH, K_2_CO_3_, *N,N*-dimethylacetamide dimethyl acetal (90%) 44-azobis(4-cyanovaleric acid) (ACVA; ≥98.0%), were all purchased from Sigma-Aldrich and used without further treatments. The organic solvents of A.R. grade were obtained from Guanghua Sci-Tech Co., Ltd. (Guangdong, China) and used as received. The CO_2_ and N_2_ gas with a purity of above 99.99% were obtained from the Jinnengda Gas Co. in Chengdu, China. The chain transfer agent (CTA), 4-cyano-4-thiothiopropylsulfanylpentanoic acid (CTPPA), was synthesized according to the previously reported procedures [19,20].

^1^H NMR spectra were recorded at 25 °C on a Bruker AV300 NMR spectrometer at 300 MHz. The chemical shifts (*δ*) are reported in parts per million (ppm) with reference to the internal standard protons of tetramethylsilane (TMS). The conductivity was determined by an FE30 conductometer (Mettler Toledo, USA) at room temperature.

### Synthesis of 4-Vinylbenzylamine (compound 1)

In a 250 mL round-bottom flask, 22.9 g 4-vinylbenzyl chloride (0.15 mol) and 27.8 g phthalimide potassium salt (0.15 mol) were dissolved into 100 mL dimethyl formamide (DMF) solution and stored at 50 °C for 4 h with stirring. The solvent was removed by reduced pressure distillation after reaction. Then the mixture was dissolved in CHCl_3_ and washed with NaOH solution (0.2 mol·L^−1^) and water successively. The raw product was concentrated and recrystallized in methanol for two times, yielding colorless transparent crystals.

In the following step, the colorless transparent crystals (~27.1 g) were dissolved into 50 mL ethanol with heating. Then 12.4 g N_2_H_4_·H_2_O (0.13 mol) was slowly added into the solution, producing white precipitations. After 90 min reaction, white solids were obtained by filtration. Then the solids were dissolved with 15 wt% KOH solution, followed by filtration. The filtrate was extracted for three times with diethyl ether and washed with 2 wt% K_2_CO_3_ aqueous solution. Colorless transparent liquid was obtained after drying with K_2_CO_3_, followed by removing solvent with rotary evaporation. ^1^H NMR (*δ*, ppm; solvent, CDCl_3_), 3.85 (–CH_2_NH_2_), 5.20–5.24, 5.70–5.74 (–CH=CH_2_), 6.66–6.75 (–CH=CH_2_), 7.2, 7.3 (–C_6_H_4_–CH=CH_2_).

### Synthesis of 4-vinylbenzyl amidine (compound 2)

6.6 g *N,N*-Dimethylacetamide dimethyl acetal (45 mmol) was added into 150 mL round-bottom flask, followed by dropping 5.0 g colorless transparent liquid obtained from last step (compound **1**) under N_2_ protection. The mixture was stirred for 15 min at room temperature and then heated to 65 °C. After 2 h reaction, the product was collected by removing side product methanol and excess *N,N*-Dimethylacetamide dimethyl acetal with rotary evaporation. ^1^H NMR (*δ*, ppm; solvent, CDCl_3_), 1.91 ((CH_3_)_2_ N– (CH_3_)C=N–), 2.95 ((CH_3_)_2_ N– (CH_3_)C=N–), 4.47–4.50 (–C_6_H_4_–CH_2_–N), 5.15–5.29 and 5.59–5.76 (CH_2_=CH–), 6.65–6.74 (CH_2_=CH–), 7.25–7.36 (–C_6_H_4_–).

### RAFT polymerization of 4-vinylbenzyl amidine

The RAFT polymerization is carried out as following typical procedure. 1.02 g 4-vinylbenzyl amidine (5.0 mmol), 14 mg CTPPA (0.05 mmol) and 3 mg initiator ACVA and 2 mL of solvent was added into a reaction tube. Then the system was deoxygenated with three freeze-thaw cycles or bubbling N_2_ for 30 min followed by keeping at 70 °C for 24 h. After the reaction, the product was checked with ^1^H NMR.

## Results and discussion

### CO_2_-responsiveness of 4-vinylbenzyl amidine

**Figure 1.**
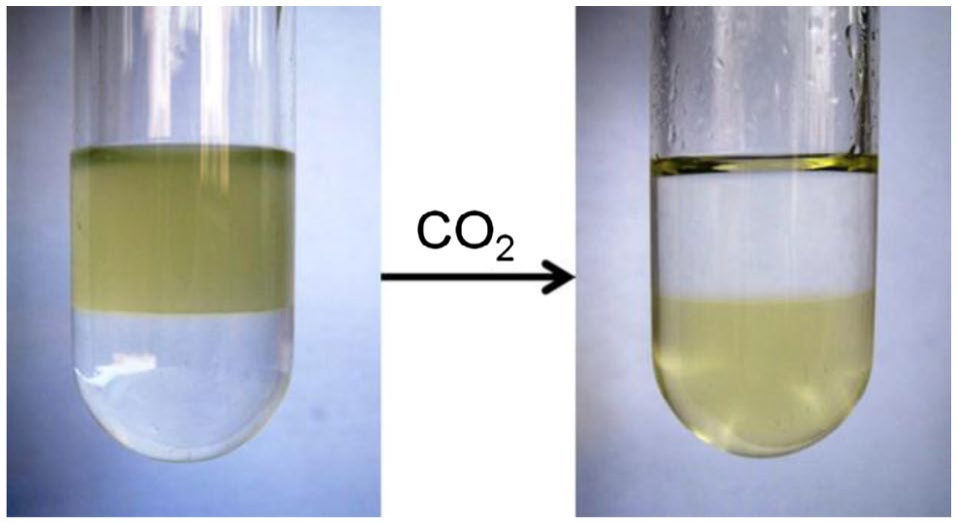
The hydrophobic to hydrophilic transformation of 4-vinylbenzyl amidine upon stimulation of CO_2_. The solvent is *n*-hexane and water (v/v = 1:1).

As above-mentioned, we previously developed CO_2_-responsive polymers by combing RAFT polymerization and ‘click’ chemistry, which is not suitable for preparation of complex copolymers, such as multi-block and star structures [8,9]. Therefore, we try to synthesize a CO_2_-responsive monomer in this work. After we confirm the chemical structure of this monomer by NMR (see experimental part), its solubility is tested, finding it can be dissolved in common organic solvent including *n*-hexane, CH_2_Cl_2_, CHCl_3_, THF, DMF, DMSO. To check out the CO_2_-sensitivity, we then investigate whether it can transform from organic solvent into water phase, meaning whether it can transition from hydrophobicity to hydrophility. As shown in Figure 1, in a bi-phase solvent of *n*-hexane and water (v/v = 1:1), several drops monomer is added into the system. The monomer is dissolved into *n*-hexane in upper phase without CO_2_ treatment, appearing as a yellow solution above water. Then CO_2_ gas is bubbled into the solution, resulting in a yellow aqueous solution in the bottom. Meanwhile, the upper *n*-hexane becomes colorless and transparent. The *n*-hexane is a non-polar solvent, this transition implies the monomer can transform from hydrophobicity into hydrophilicity upon CO_2_ stimulation.

**Figure 2.**
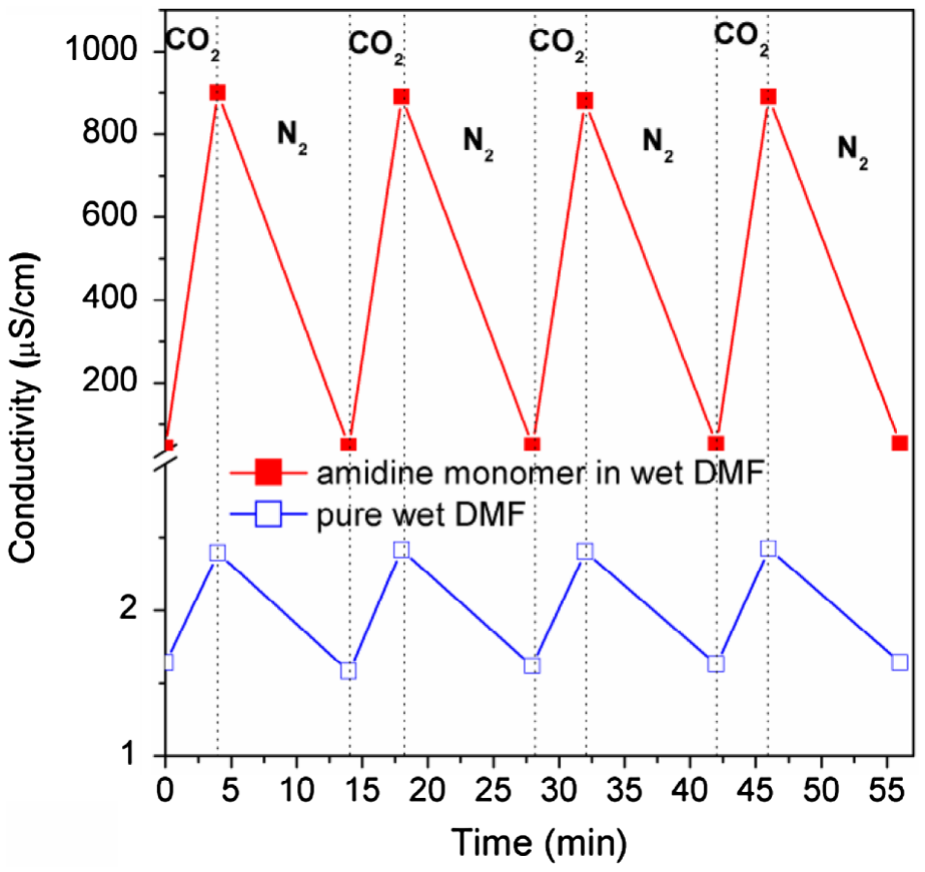
Conductivity variation of 4-vinylbenzyl amidine in wet DMF solution (~1 v% water) when CO_2_ and N_2_ is alternatively bubbling.

To confirm the switchability of this monomer, we monitor the conductivity variation of its wet DMF solution during CO_2_ is bubbling and removing alternately. As shown in Figure 2, the conductivity of the monomer solution jumps from 43.0 to 900.1 μS cm^−1^ after bubbling CO_2_ for 4 min. Subsequently, it drops back to around 45.6 μS cm^−1^ after removing CO_2_ by N_2_ treatment. Furthermore, this jumping-dropping cycle can be repeated for four times without significant attenuation, indicating the CO_2_-responsiveness of this monomer is reversible. As a comparison, the conductivity of wet DMF solution without monomer inside only increases from 1.6 to 2.4 μS cm^−1^ under the stimulation of CO_2_, which further proves the switchable transition of the monomer under stimulation of CO_2_.

**Figure 3.**
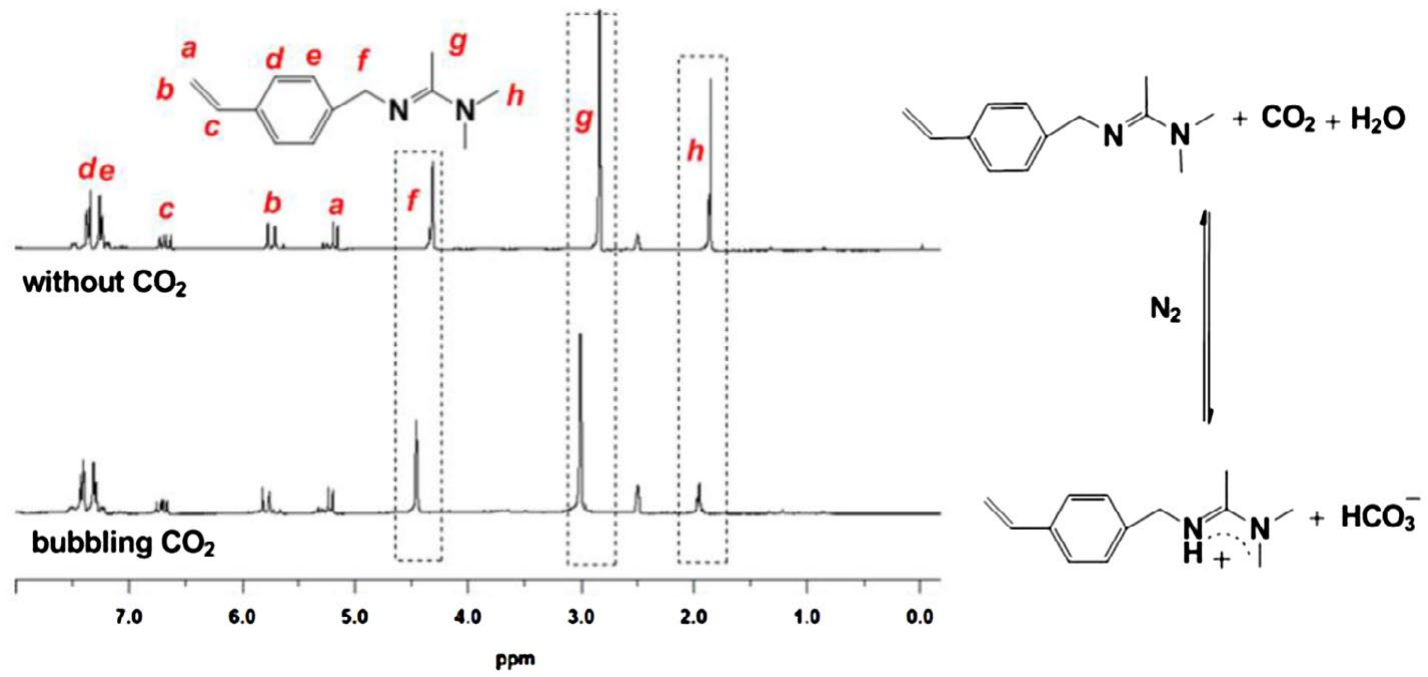
^1^H NMR spectra of 4-vinylbenzyl amidine before and after reaction with CO_2_ and the reaction equation of amidine and CO_2_. The solvent is wet DMSO-*d*6.

Beside the conductivity, we further investigate the reaction of the monomer with CO_2_ by ^1^H NMR characterization (Figure 3). The monomers were dissolved into wet DMSO-*d*6 and characterized by ^1^H NMR before and after the treatment of CO_2_. By comparing the main proton peak around the amidine group, one can easily indentify the significant chemical shift, i.e. *f*[–C_6_H_4_–CH_2_–N=C(CH_3_)–], *g*[N=C(CH_3_)–N(CH_3_)_2_], *h*[N=(CH_3_)C–N(CH_3_)_2_] increases from 4.32, 2.84, 1.85 to 4.46, 3.01, 1.95 ppm, respectively. This result proves the protonation of the monomer after reaction with CO_2_. The protonation reaction is shown in Figure 3, i.e. the amidine react with CO_2_ and water producing bicarbonate. Actually, similar reaction of amidine and CO_2_ in the presence of water has been recognized by other researchers [21]. This protonation produces a compound with positive charge [5] which also accounts for why the monomer can transform from hydrophobic to hydrophilic state under stimulation of CO_2_.

**Figure 4.**
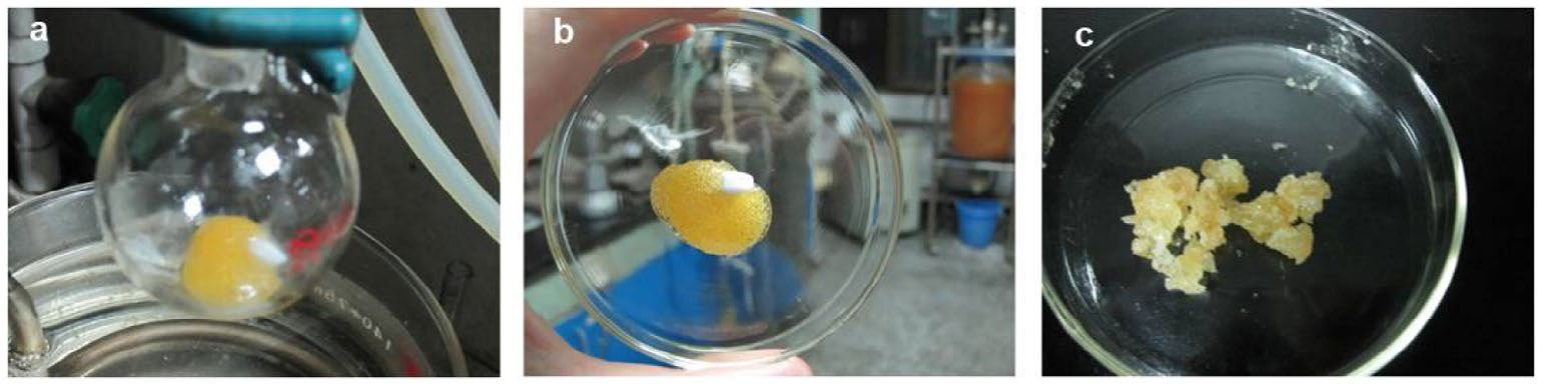
Snapshots of the RAFT polymerization product of 4-vinylbenzyl amidine. (a) product after reaction; (b) product in watch-glass; (c) product after drying.

**Figure 5.**
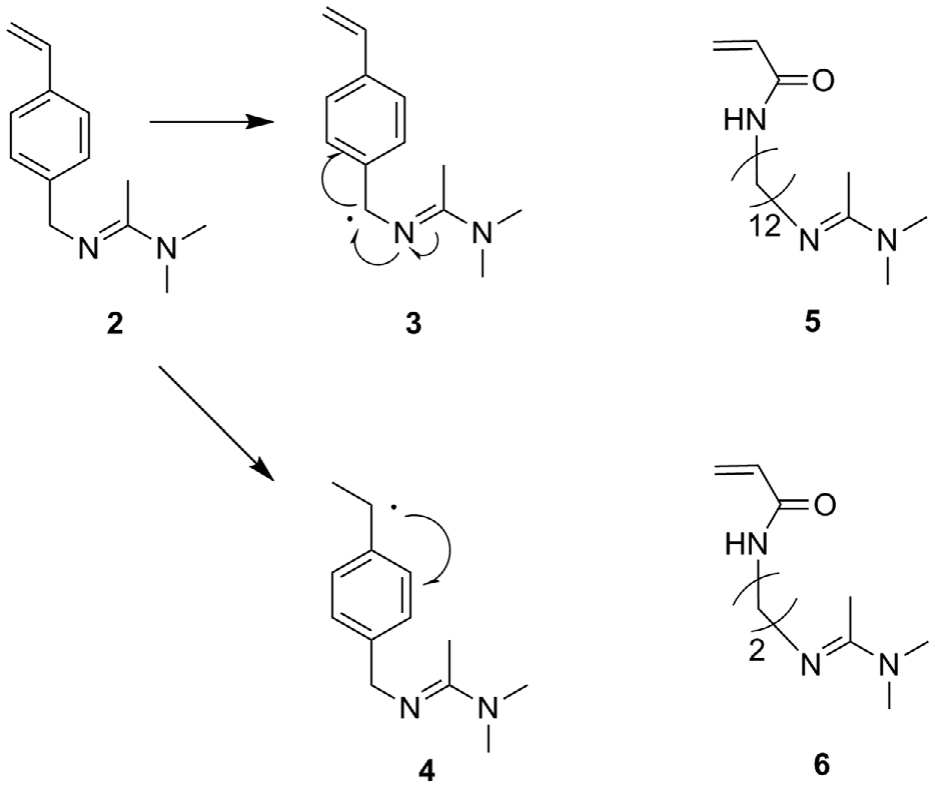
Illustration of possible radical structures of 4-vinylbenzyl amidine in radical polymerization (structure **3** and **4**) and reported amandine monomers by the groups of Yuan (compound **5**, Ref. [11]) and Yung (compound **6**, Ref. [14]).

### Polymerization of 4-vinylbenzyl amidine

**Scheme 2.**
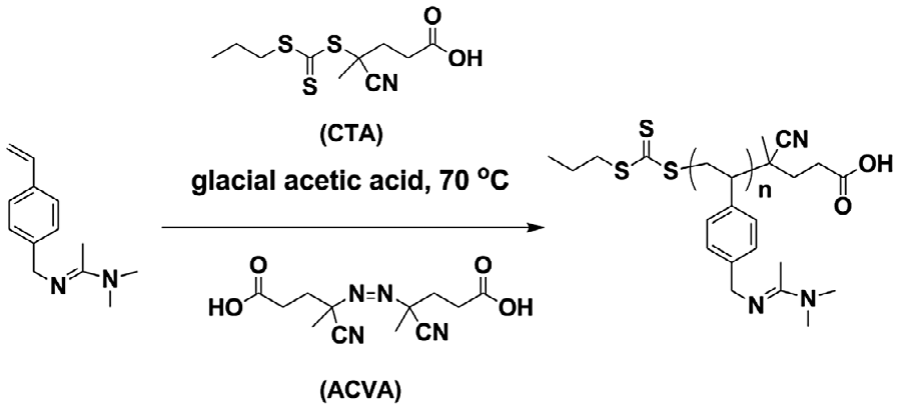
RAFT polymerization of 4-vinylbenzyl amidine.

After confirm the chemical structure and CO_2_-responsive feature of the monomer, we try to carry out the RAFT polymerization (Scheme 2). However, we get nothing when the reaction is done in organic solvent, neither in THF nor DMF. The reason, in our mind, might be the basicity of the amidine group (will discuss further). Long and his coworkers [22] reported RAFT polymerization of 4-vinylimidazole in glacial acetic acid (HAc), which is another basic monomer that is difficult to conduct controlled polymerization. Inspired by this discovery, we perform RAFT polymerization of 4-vinylbenzyl amidine using HAc as solvent (Scheme 2). The reaction conditions are illustrated in Table 1.

**Table 1.** Reaction conditions of RAFT polymerization of 4-vinylbenzyl amidine.

No.	De-oxygenization	Solvent	Initiator/CTA^c^	Product
1	Freezing-thaw	HAc/DMF=1:1^a^	0.2:1	None
2	Freezing-thaw	HAc^b^	0.2:1	None
3	Freezing-thaw	HAc	1:1	None
4	Bubbling N_2_ 30 min	HAc	0.2:1	None
5	Bubbling N_2_ 30 min	HAc	1:1	Cross-linked gel

^a^ Volume ratio.

^b^ Glacial acetic acid.

^c^ Molar ratio.

At first, we tried to polymerize 4-vinylbenzyl amidine in mixed solvent of DMF and HAc, but failed. Then various efforts were made, including utilization of pure HAc, increasing of the initiator ratio and optimization of de-oxygenization method (Table 1). Finally, we find this monomer can be polymerized in conditions: (1) pure HAc, (2) initiator/CTA = 1, (3) bubbling N_2_ for 30 min to remove oxygen (entry 5, Table 1). Nevertheless, the product appears as cross-linked gel with yellow color rather than polymer dissolved in solution (Figure 4). This gel cannot be dissolved into any solvents. Further improvements of this polymerization are still progressing in our group. Interestingly, the de-oxygenization method really affects the success of this reaction, if comparing entry 3 and 5 in Table 1. The reason may be ascribed to the highly volatile feature of HAc, which make it firstly filling the reaction tube in vacuum that restrains the discharge of oxygen in solution.

Then we come back to the problem, why the 4-vinylbenzyl amidine cannot be polymerized in neutral state without acetic acid? In the presentence of initiator, two kinds of radicals are possible produced; structure **3** and **4** (Figure 5). The radical **3** is more stable then **4**, because it forms a huge conjugated structure combining benzene ring and the donor amidine group. That means the 4-vinylbenzyl amidine transforms into radical **3** in organic reaction system, hindering the polymerization we desired. When the amidine groups are protonated by acetic acid, the possible huge conjugated structure **3** cannot form anymore and the polymerization occurs through the same mechanism of the radical polymerization of polystyrene. It should be point out that the reported amidine monomers (compounds **5** and **6** in Figure 5) can be polymerized through a radical mechanism in their natural state because they have no concerning to form stable structure like radical **3**.

## Conclusions

In conclusion, a CO_2_-responsive monomer, 4-vinylbenzyl amidine, was designed and synthesized in this work. It transforms from hydrophobic to hydrophilic state under the stimulation of CO_2_, and this transition is reversible. This monomer can be polymerized by RAFT technique in glacial acetic acid, yielding a gel-like homo-polymer. The results of this work provide another choice for preparing new CO_2_ responsive polymers and pave a way to the development of novel CO_2_-sensitive ‘smart’ system.

## Disclosure statement

No potential conflict of interest was reported by the authors.

## Funding

This work was financially supported by the National Natural Science Foundation of China [grant number: 21273223]; and the open funding of State Key Laboratory of Polymer Materials Engineering [grant number: sklpme2014-2-06].
